# Ataque da inteligência Artificial! A Crescente Importância da Doença de Chagas

**DOI:** 10.36660/abc.20260409

**Published:** 2026-06-19

**Authors:** Roberto Coury Pedrosa

**Affiliations:** 1 Universidade Federal do Rio de Janeiro Instituto do Coração Edson Saad Departamento de Cardiologia Rio de Janeiro RJ Brasil Departamento de Cardiologia, Hospital Universitário Clementino Fraga Filho - Instituto do Coração Edson Saad - Universidade Federal do Rio de Janeiro, Rio de Janeiro, RJ – Brasil

**Keywords:** Doença de Chagas, Inteligência Artificial, Eletrocardiografia, Saúde Pública

A doença de Chagas (DC) é uma condição crônica com sequelas principalmente cardíacas que podem persistir e piorar muitos anos após a infecção inicial, com sério impacto nos sistemas de saúde. A Organização Pan-Americana da Saúde¹ estimou que 90% das novas infecções não são diagnosticadas e cerca de 70% dos infectados desconhecem sua condição. Além disso, menos de 1% da população estimada em risco foi testada.² Como resultado, isso leva a um atraso no diagnóstico, o que aumenta a morbidade e a mortalidade decorrentes de suas complicações, destacando a clara necessidade de ampliar o acesso ao diagnóstico sob a perspectiva da saúde pública. Para mitigar esse impacto, a DC foi incluída nos Objetivos de Desenvolvimento do Milênio e nos Objetivos de Desenvolvimento Sustentável da Agenda 2030 para o Desenvolvimento Sustentável (ONU, 2018).

O padrão ouro para o diagnóstico da DC é o teste sorológico para anticorpos contra o *T. cruzi.*³ O maior desafio na DC continua sendo a previsão de quais pacientes desenvolverão a forma cardíaca, uma das principais causas de insuficiência cardíaca, especialmente em áreas rurais do Brasil. Notavelmente, a eletrocardiografia é uma ferramenta acessível e de baixo custo que pode detectar sinais precoces de comprometimento cardíaco na DC.³ Nos últimos anos, modelos de inteligência artificial (IA) baseados no eletrocardiograma (ECG)^4^ têm demonstrado capacidade de diagnosticar condições cardíacas com precisão; muitos foram treinados e testados principalmente com dados de países de alta renda. Além disso, esses modelos foram avaliados retrospectivamente em grandes conjuntos de dados históricos, com relativamente poucos estudos prospectivos de validação externa. Essas lacunas na pesquisa em ECG com IA precisam ser abordadas.

Nesta edição da ABC, Fonseca et al.^5^ apresentam os resultados de seu estudo transversal prospectivo de validação externa, que busca preencher essas duas lacunas. A avaliação foi conduzida como parte de uma iniciativa multicêntrica de triagem cardiovascular. Os pesquisadores testaram um algoritmo de ECG pré-existente aprimorado por IA (modelo de *deep learning*) combinado com dados clínicos (residência prévia em áreas infestadas por triatomíneos, condições precárias de moradia e histórico familiar de DC) para o diagnóstico da doença em uma região altamente endêmica da América Latina, em indivíduos sem suspeita clínica da doença e em um contexto de saúde com poucos recursos. A validação das predições do ECG baseado em IA foi realizada por meio de testes sorológicos positivos para anticorpos contra *T. cruzi*. Foram incluídos 1.115 adultos submetidos a ECG padronizado de 12 derivações. O algoritmo identificou 121 indivíduos (10,9%), correspondendo a uma prevalência de 7,8% (IC 95%: 6,2–9,6). Desses, 112 foram submetidos a testes sorológicos; 13 testaram positivo, todos do grupo suspeito de DC com IA positiva. Nenhum caso soropositivo foi identificado no grupo controle. Os autores relatam métricas de desempenho impressionantes, com sensibilidade de 100% (IC 95%: 69–100), especificidade de 40% (IC 95%: 30–50), valor preditivo negativo de 100% (IC 95%: 91–100) e valor preditivo positivo de 12% (IC 95%: 11–14), com uma razão de chances diagnósticas de 6,6. Notavelmente, a área sob a curva ROC (característica de operação do receptor) e a omissão da medida de desempenho F1 devido ao desequilíbrio dos dados não foram relatadas.^6^ Segundo os autores, suas descobertas demonstram que a integração de um algoritmo de ECG aprimorado por IA com perguntas clínicas básicas pode identificar com precisão casos de DC soropositivos.

O uso de IA para o diagnóstico de DC é certamente importante. No entanto, seu uso no diagnóstico da forma cardiaca é ainda mais relevante, visto que, em países tradicionalmente endêmicos, a incidência de DC tem diminuído gradualmente, em favor de um aumento da forma cardíaca. Notavelmente, no presente estudo, os autores não exploraram adequadamente o consenso de que a alteração no ECG reflete a forma cardíaca na DC.^3^

Portanto, esses resultados devem ser interpretados nesse contexto, e cautela deve ser aplicada ao extrapolar esses dados para outros cenários. Não obstante, esta avaliação prospectiva do ECG com IA em um ambiente de saúde com recursos limitados por Fonseca et al.^5^ é uma adição bem-vinda à crescente literatura sobre IA-ECG, e mais estudos prospectivos como este são incentivados.
