# Supplementary material for: Ataque da inteligência Artificial! A Crescente Importância da Doença de Chagas
Source: Arq Bras Cardiol. 2026 Jun 19;123(5):e20260409. [Article in Portuguese] doi: 10.36660/abc.20260409 (PMC13399161; doi:10.36660/abc.20260409)
Supplement: Translation

# Translation: Artificial Intelligence Attack! The Rising Importance of Chagas Disease

**DOI:** 10.36660/abc.20260409i

**Authors:** Roberto Coury Pedrosa

**Affiliations:** 1 Rio de Janeiro RJ Brazil coury@hucff.ufrj.br Departamento de Cardiologia, Hospital Universitário Clementino Fraga Filho – Instituto do Coração Edson Saad – Universidade Federal do Rio de Janeiro, Rio de Janeiro, RJ – Brazil

**Keywords:** Chagas disease, Artificial Intelligence, Electrocardiography, Screening, Public Health

Chagas disease (Chd) is a chronic condition with primarily cardiac sequelae that can persist and worsen many years after the initial infection, with a serious impact on healthcare systems. The PAHO^1^ estimated that 90% of new infections go undiagnosed, and about 70% of those infected are unaware of their condition. Additionally, less than 1% of the estimated population at risk has been tested.^2^ As a result, this leads to a delay in diagnosis, which increases morbidity and mortality from its complications, highlighting a clear need to expand access to diagnosis from a public health perspective. To mitigate this impact, Chd was included in the Millennium Development Goals and the Sustainable Development Goals of the 2030 Agenda for Sustainable Development (UN, 2018).

The gold standard for diagnosing Chd is serological testing for *T. cruzi* antibodies.^3^ The biggest challenge in Chd remains predicting which patients will develop the cardiac form, a leading cause of heart failure, especially in rural areas of Brazil. Notably, electrocardiography is an accessible and low-cost tool that can detect early signs of cardiac involvement in Chd.^3^ In recent years, AI-ECG (Electrocardiogram with artificial intelligence) models^4^ have been shown to diagnose cardiac conditions accurately; many have been trained and tested primarily on data from high-income countries. Furthermore, these models have been evaluated retrospectively on large historical datasets, with relatively few prospective external validation studies. These gaps in AI-ECG research need to be addressed.

In this issue of ABC, Fonseca et al.^5^ report results from their external validation prospective cross-sectional study, which seeks to address both these gaps. The evaluation was conducted as part of a multicentre cardiovascular screening initiative. They tested a pre-existing AI-enhanced ECG algorithm (deep learning model) combined with clinical data (previous residence in triatomine-infested areas, poor housing conditions, and family history of Chd) for Chd diagnosis in a highly endemic region of Latin America, among individuals without clinical suspicion of Chd and a low-resource health care setting, The validation of the AI-ECG predictions was against positive serological testing for *T. cruzi* antibodies. They enrolled 1,115 adults who underwent standardised 12-lead ECG acquisition. The algorithm flagged 121 individuals (10.9%), corresponding to a prevalence of 7.8% (95% CI: 6.2–9.6). Of these, 112 underwent serological testing; 13 tested positive, all from the AI-positive suspected Chd group. No seropositive cases were identified among controls. The authors report impressive performance metrics, with a sensitivity was 100% (95% CI: 69–100), specificity 40% (95% CI: 30–50), negative predictive value 100% (95% CI: 91–100), and a positive predictive value 12% (95% CI: 11–14), with a diagnostic odds ratio of 6.6. Notably, the area under the receiver operating characteristic (ROC) curve, and the omission of the F1 score performance measure due to data imbalance were not reported.^6^ According to the authors, their findings demonstrate that integrating an AI-enhanced ECG algorithm with basic clinical questions can accurately identify seropositive Chd cases.

The use of AI for the diagnosis of Chd is certainly important. Still, its use in the diagnosis of CC is even more relevant, given that in traditionally endemic countries the incidence of Chd has been gradually decreasing, in favour of an increase in the cardiac form. Notably, in the present study, the authors did not adequately explore the consensus that the ECG-altered reflects the cardiac form in Chd.^3^

Therefore, these results should be interpreted in this context, and caution must be applied in extrapolating these data to other settings. Nonetheless, this prospective evaluation of AI-ECG in a resource-limited health care setting by Fonseca et al.^5^ is a welcome addition to the growing AI-ECG literature, and further such prospective studies are encouraged.
